# *Rice Flowering Locus T 1* plays an important role in heading date influencing yield traits in rice

**DOI:** 10.1038/s41598-017-05302-3

**Published:** 2017-07-07

**Authors:** Yu-Jun Zhu, Ye-Yang Fan, Kai Wang, De-Run Huang, Wen-Zhen Liu, Jie-Zheng Ying, Jie-Yun Zhuang

**Affiliations:** 1 State Key Laboratory of Rice Biology and Chinese National Center for Rice Improvement, China National Rice Research Institute, Hangzhou, 310006 China; 2 Yuanlongping High-Tech Agriculture Co., Ltd., Changsha, 410001 China

## Abstract

Important role of flowering genes in enhancing grain productivity in rice has become well recognized for a number of key genes regulating the florigen production, but little has been known for the two florigen genes themselves. In this study, pleiotropism of *Rice Flowering Locus T 1* (*RFT1*), one of the two florigen genes in rice, was firstly evaluated using near isogenic lines (NILs) carrying *RFT1* alleles from the *indica* rice cultivars Zhenshan 97 (ZS97) and Milyang 46, respectively, and then determined by transformation of the *RFT1* ^ZS97^ allele into a *japonica* rice variety, Zhonghua 11. The *RFT1* ^ZS97^ allele was shown to delay heading and increase plant height, grain weight, grain number and grain yield, indicating that *RFT1* plays an important role in the growth and development of rice. This study has also validated the potential of using a new type of genetic resource, sequential residual heterozygotes (SeqRHs), for QTL fine-mapping. A step-by-step approach was employed for SeqRHs identification, NIL development and QTL fine-mapping. The heterozygous segments and candidate QTL regions were gradually narrowed down. Eventually, the QTL region was delimited to a 1.7 kb region containing a single gene.

## Introduction

Rice (*Oryza sativa* L.) is the staple food for half of the world’s population. Among strategies for developing rice varieties with higher yield potential and greater yield stability is the identification and introduction of beneficial genes^1^. In the past two decades, great efforts have been made on the characterization of quantitative trait loci (QTLs) underlying complex traits in rice. Following extensive mapping using primary populations, a large number of QTLs were documented in databases such as the Gramene (http://archive.gramene.org/qtl/) and the main attention has been moved to the validation, fine mapping and cloning. More and more QTLs were isolated as a single gene, providing useful targets for marker-assisted selection and molecular design breeding^2–4^. Usually, a limited number of traits were analyzed in the cloning studies, but a gene is in fact associated with many more traits^5^ due to the ubiquity of pleiotropy^6^. A better understanding of pleiotropism of the cloned QTLs is of great importance for designing a molecular breeding strategy and making an efficient selection criterion for the target traits and genes.

Cloning of QTLs for flowering time in rice has played a fundamental role in understanding the complex gene network for flowering regulation^7, 8^ and provided exceptional evidences for the pleiotropism of flowering genes in controlling yield traits^9–20^. The first cloned flowering QTL found to play a crucial role for increasing the grain productivity of rice is *Ghd7* ^9^. In the regulatory networks for the florigens in rice, *Ghd7* is not only one of the two key genes involved in the evolutionarily unique *Ehd1*-*RFT1* pathway^7^, but also a pivotal gene connecting the *Ehd1*-*RFT1* pathway and the evolutionarily conserved *Hd1-Hd3a* pathway^21^. The role of *Ghd7* as an important gene determining yield potential of rice has become better and better understood^10–13^, along with similar finding for other key flowering genes. Included are *DTH8*/*Ghd8* ^13–15^ and *OsPRR37*/*Ghd7.1*/*DTH7*/*Hd2* ^12, 16–18^ that are major regulators of the *Ehd1*-*RFT1* and *Hd1*-*Hd3a* pathways^8^, respectively, and the two central genes *Hd1* ^11, 13, 19, 20^ and *Ehd1* ^20^ themselves.

While the important role of flowering genes in determining the yield potential of cultivated rice has become well recognized for a number of key genes regulating the florigen production, little has been known for *Hd3a* and *RFT1*, the two florigen genes immediately trigger and control flowering in rice. In the present study, the effect of *RFT1* was firstly evaluated using near isogenic lines (NILs) carrying *RFT1* alleles from the *indica* rice cultivars Zhenshan 97 (ZS97) and Milyang 46 (MY46), respectively, and then determined by transformation of the *RFT1* ^ZS97^ allele into a *japonica* rice variety, Zhonghua 11 (ZH11). The *RFT1* ^ZS97^ allele was shown to delay heading and increase plant height, grain weight, grain number and grain yield. The results indicate that *RFT1* play an important role in the growth and development of rice.

Another goal of this study is attempting to locate a QTL into a single-gene region by using a new type of genetic resource, sequential residual heterozygotes (SeqRHs). One series of SeqRHs contains a number of residual heterozygotes (RHs), of which the heterozygous segments are overlapped in sequence based on the physical positions and jointly cover the entire candidate QTL region^22^. Targeting at a QTL region previously detected on the short arm of chromosome 6, a step-by-step approach was employed for the identification of SeqRHs, development of NILs and fine mapping of the QTL. The heterozygous segments and candidate QTL regions were gradually narrowed down. Eventually, the QTL region was delimited to a 1.7-kb region containing a single gene locus, *RFT1*.

## Results

### Progressive narrowing down of the candidate QTL region using SeqRHs

**Figure 1:**
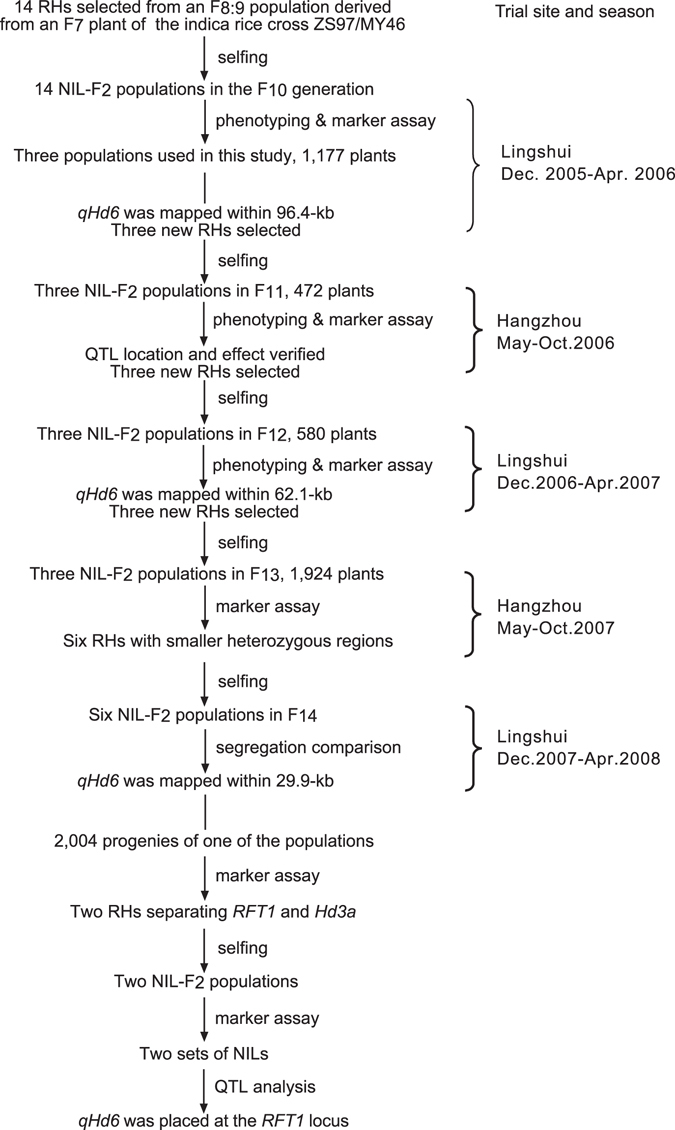
The procedure used for fine-mapping *qHd6* in this study. A three-step procedure, selecting RHs overlapped in the candidate QTL region – constructing NIL-F_2_ populations – performing QTL analysis, was repeated until the QTL was placed at the *RFT1* locus. NIL, near isogenic line. RH, residual heterozygote.

A three-step procedure, selecting RHs overlapped in the candidate QTL region – constructing NIL-F_2_ populations – performing QTL analysis, was repeated until the QTL was placed at the *RFT1* locus (Fig. 1). We have previously constructed an F_8:9_ population segregated in a 7.3-Mb region on the short arm of chromosome 6 from an F_7_ plant of the *indica* rice cross ZS97/MY46^23^. Fourteen F_9_ plants carrying overlapped heterozygous segments jointly covering the entire 7.3-Mb region were selected (Supplementary Fig. S1 and Table S1). The resultant 14 NIL-F_2_ populations were grown in Lingshui from Dec. 2005 to Apr. 2006 under the natural short-day (NSD) conditions. Three of them were used in the present study.

**Figure 2:**
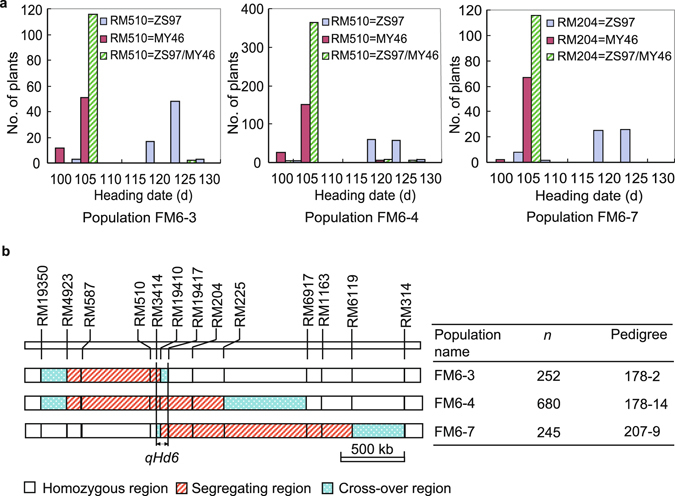
Heading date segregation of the three NIL-F_2_ populations firstly used in this study. (**a**) Distribution of heading date and its association with the three genotypic groups in each population. (**b**) Segregating regions of the three populations and the 96.4-kb region mapped for *qHd6*. The three populations are each constructed from selfed seeds of an RH plant in F_9_ which is a progeny of an F_7_ plant of the *indica* rice cross ZS97/MY46. Pedigree refers to the pedigree of the residual heterozygote that was selfed to produce the NIL-F_2_ population, in which the first and second numbers are the Plant No. in F_8_ and F_9_, respectively. *n*, number of plants.

Heading date (HD) of the three NIL-F_2_ populations was discretely distributed and co-segregated with DNA markers in the target region (Fig. 2a). These results indicate that a QTL for HD was located in the common segregating region of the three populations. The QTL, designated as *qHd6*, was delimitated within the region flanked by simple sequence repeat (SSR) markers RM3414 and RM19417 (Fig. 2b). This region corresponds to a 96.4-kb region in the Nipponbare genome, containing the *Flowering Locus T* (*FT*) gene *Hd3a* that has been cloned at the time^24^ and its paralog *Twin Sister of FT* that was later characterized and known as *Rice Flowering Locus T 1* (*RFT1*)^25^. As compared with MY46, the ZS97 allele delayed heading by 9.0−9.7 d under NSD conditions in Lingshui (Table 1).

**Table 1:** Heading date of two homozygous genotypes differing in the *qHd6* region.

Population Name	Trial site	Day length	Heading date (d)				*A* ^a^
			*qHd6* ^ZS^	*n*	*qHd6* ^MY^	*n*	
FM6-3	Lingshui	Natural short day	121.3 ± 2.3^****^	68	101.8 ± 1.2	63	9.7
FM6-4	Lingshui	Natural short day	121.1 ± 2.1^****^	122	101.9 ± 1.1	179	9.6
FM6-7	Lingshui	Natural short day	120.6 ± 1.5^****^	51	102.7 ± 1.1	69	9.0
SF6-5	Hangzhou	Natural long day	100.4 ± 2.0^****^	19	80.9 ± 0.9	16	9.7
SF6-9	Hangzhou	Natural long day	100.1 ± 3.2^****^	29	81.8 ± 1.5	68	9.2
TF-15	Lingshui	Natural short day	118.2 ± 1.9^****^	53	103.5 ± 2.5	52	7.3
NIL-1	Phytotron	Short day	72.3 ± 1.8^****^	10	60.5 ± 1.7	10	5.9
NIL-1	Phytotron	Long day	90.3 ± 2.6^****^	10	76.0 ± 2.2	10	7.1
NIL-2	Phytotron	Short day	72.8 ± 1.8^****^	10	60.2 ± 1.9	10	6.3
NIL-2	Phytotron	Long day	90.2 ± 2.2^****^	10	76.2 ± 1.9	10	7.1

*qHd6* ^ZS^ and *qHd6* ^MY^ are near isogenic lines carrying homozygous *qHd6* alleles from ZS97 and MY46, respectively. The heading date is presented as mean ± *sd*. *n*, number of plants. ^****^The difference between *qHd6* ^ZS^ and *qHd6* ^MY^ is significant at *P* < 0.0001 using Student’s *t*-test. ^a^ *A*, additive effect of replacing a MY46 allele with a ZS97 allele.

**Figure 3:**
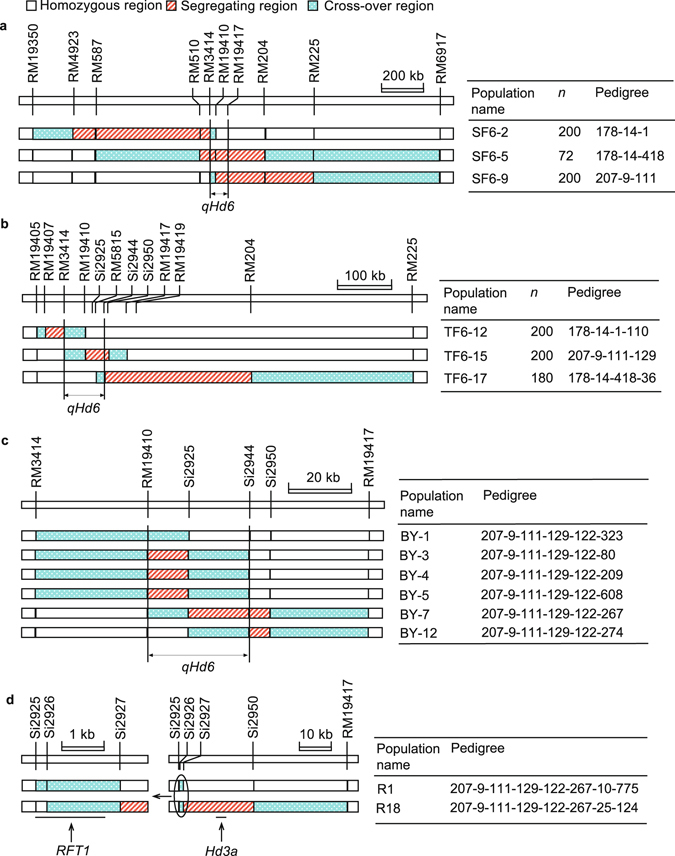
Segregating regions of four sets of NIL-F_2_ populations. (**a**) Three populations in F_11_ used to validate the location and effect of *qHd6* disclosed in the previous generation. (**b**) Three populations in F_12_ used to delimitate *qHd6* into a 62.1-kb region. (**c**) Six populations in F_14_ used to delimitate *qHd6* into a 29.9-kb region. (**d**) Two populations in F_16_ used to place *qHd6* at the *RFT1* locus and to construct NILs for analyzing the *RFT1* pleiotropism. Pedigree refers to the pedigree of the residual heterozygote that was selfed to produce the NIL-F_2_ population, in which the first number is the Plant No in F_8_, followed by those in later generations up to F_10_, F_11_, F_13_ and F_15_ in (**a,b,c** and **d**), respectively. *n*, number of plants.

Three new RHs were selected. The resultant NIL-F_2_ populations were grown in Hangzhou from May to Oct. 2006 under the natural long-day (NLD) conditions. While SF6–2 showed no segregation on HD, the SF6-5 and SF6-9 populations were discretely distributed with the ZS97 allele delaying heading by 9.7 and 9.2 d, respectively (Table 1). As shown in Fig. 3a, the 96.4-kb location of *qHd6* mapped in the previous season was included in the common segregating region of SF6-5 and SF6-9. Since the effects detected were consistent under the NLD conditions in Hangzhou and NSD conditions in Lingshui, the gene responsible for *qHd6* should be a gene without photoperiod sensitivity.

Again, three new RHs were selected. The resultant NIL-F_2_ populations were grown in Lingshui from Dec. 2006 to Apr. 2007. HD was not segregated in populations TF6-12 and TF6-17, but it was discretely distributed in TF6-15 with the ZS97 allele delaying heading by 7.3 d (Table 1). Thus, *qHd6* was delimitated to a 62.1-kb region flanked by SSR marker RM3414 and InDel marker Si2944 by excluding the segregating regions in TF6-12 and TF6-17 (Fig. 3b). The photoperiod response of *qHd6* was also tested in phytotron using two NIL pairs selected from TF6-15.The ZS97 allele delayed heading by 6.1 and 7.1 d under the short-day (SD) and long-day (LD) conditions, respectively (Table 1), confirming that this QTL is insensitive to photoperiod.

Later, six new NIL-F_2_ populations with smaller and overlapped segregating regions were constructed. Segregation on HD was found in all the populations except BY-1 and BY-12, thus *qHd6* was mapped within a 29.9-kb region flanked by RM19410 and Si2944 (Fig. 3c). Then, two RHs were selected for separating *RFT1* and *Hd3a*. In the two NIL-F_2_ populations derived, R1 was segregated for the *RFT1* gene marker Si2926 and homozygous in the *Hd3a* region, whereas R18 was homozygous at Si2926 but segregated in the *Hd3a* region (Fig. 3d). As described in next section, *qHd6* was placed at the *RFT1* locus by testing NILs derived from the two populations.

### Pleiotropism of *RFT1* on heading date, plant height and yield traits

In the NIL-F_2_ populations R1 and R18, non-recombinant plants were identified based on marker genotypes. In each population, 30 homozygous ZS97 plants, 60 heterozygous plants and 30 homozygous MY46 plants were selected. The resultant F_3_ families were tested in Hangzhou in 2010. Traits measured included HD, plant height (PH), number of panicles per plant (NP), number of grains per panicle (NGP), 1000-grain weight (TGW), and grain yield per plant (GY). In R1 which was segregated at a single marker locus, Si2926 for the *RFT1* gene, significant phenotypic difference among the three genotypic groups was detected for all the traits except NP (Table 2). Replacing of a MY46 allele with a ZS97 allele resulted in the increases of 15.61 d for HD, 12.69 cm for PH, 18.36 for NGP, 0.80 g for TGW g, and 3.70 g for GY. In R18 which was not segregated at Si2926 but segregated for *Hd3a*, significant effects were only detected for HD and PH, showing low effects of 0.96 d and 1.75 cm with the enhancing allele derived from MY46. Obviously, *qHd6* was segregated in the R1 population but not in R18.

**Table 2:** QTL analysis using two NIL-F_2:3_ populations.

Population and segregating region	Trait	Phenotypic mean			*P*	*A* ^a^	*D* ^b^	*D*/[*A*] ^c^	*R* ^*2*^(%)^d^
		NIL^ZS97^	NIL^MY46^	NIL^H^					
Population R1 segregated at Si2926 for *RFT1*	HD (d)	108.08	76.87	86.12	<0.0001	15.61	−6.35	−0.41	93.3
	PH (cm)	116.38	91.00	98.10	<0.0001	12.69	−5.59	−2.27	82.3
	NP	9.82	10.00	9.82	0.6330				
	NGP	130.44	93.71	113.99	<0.0001	18.36	1.92	0.10	55.7
	TGW (g)	29.31	27.72	28.56	<0.0001	0.80	0.04	0.06	48.0
	GY (g)	30.13	22.73	26.70	<0.0001	3.70	0.27	0.07	26.7
Population R18 segregated in Si2926–Si2950 containing *Hd3a*	HD (d)	76.75	78.67	77.40	<0.0001	−0.96	−0.31	−0.33	30.5
	PH (cm)	90.92	94.42	92.51	<0.0001	−1.75	−0.16	−0.09	19.8
	NP	9.61	9.90	9.88	0.1331				
	NGP	103.12	102.75	101.16	0.1178				
	TGW (g)	27.52	27.46	27.42	0.4179				
	GY (g)	23.95	24.72	24.28	0.2293				

NIL^ZS^ and NIL^MY^ are near isogenic lines carrying homozygous alleles from ZS97 and MY46, respectively. NIL^H^ are segregating lines derived from the ZS97/MY46 heterozygote. Each population consisted of 30 NIL^ZS^ lines, 60 NIL^H^ lines, and 30 NIL^MY^ lines. They were grown in Hangzhou in 2010 using a randomized complete block design with two replications. HD, heading date. PH, plant height. NP, number of panicles per plant. NGP, number of grains per panicle. TGW, 1000-grain weight. GY, grain yield per plant. ^a^ *A*, additive effect of replacing a MY46 allele with a ZS97 allele. ^b^ *D*, dominance effect. ^c^ *D*/[*A*], degree of dominance. ^d^ *R* ^*2*^, proportion of phenotypic variance explained by the QTL effect.

Sequence comparison between the *RFT1* alleles from ZS97 (NCBI GenBank No. JX473738.1) and MY46 (NCBI GenBank JX473737.1) identified 11 single nucleotide polymorphisms including two in extron 1 and nine in extron 4 (Supplementary Fig. S2). As compared with MY46, substitution of six amino acids was found in ZS97. They are V31A, E105K, S144N, R146K, N147D and T160A (Supplementary Fig. S3), among which the E105K substitution has been reported to cause a defeat in promoting flowering^26^. These results indicate that *RFT1* is the gene responsible for *qHd6*.

Pleiotropism of *RFT1* on HD and yield traits was also tested in two more years in Hangzhou, using 20 homozygous ZS97 lines and 20 homozygous MY46 lines of the R1 population. Significant effects with the enhancing allele derived from ZS97 were detected for HD, NGP, TGW and GY in both years (Table 3). The effect stayed to be large on HD, TGW and GY, but it became much smaller on NGP. In addition, a small effect with marginal significance (*P* = 0.0259) was observed for NP in 2011, in which the enhancing allele was derived from MY46. The three-year’s trial clearly showed that *RFT1* have a large effect on the grain productivity in rice and its influence on the component traits of grain yield may varied considerably across different environments.

**Table 3:** Validation of the effect of *RFT1* on HD and yield traits.

Year	Trait	Phenotypic mean		*P*	*A* ^a^	*R* ^*2*^(%)^b^
		NIL^ZS^	NIL^MY^			
2011	HD (d)	104.35	75.90	<0.0001	14.23	99.7
	NP	12.02	12.82	0.0259	−0.40	8.3
	NGP	107.83	98.24	0.0006	4.80	17.9
	TGW (g)	30.08	28.80	<0.0001	0.64	36.0
	GY (g)	30.87	24.08	<0.0001	3.39	45.5
2013	HD (d)	112.43	89.17	<0.0001	11.63	99.2
	NP	9.80	10.05	0.2198		
	NGP	123.51	116.92	0.0033	3.30	8.1
	TGW (g)	27.90	26.00	<0.0001	0.95	79.8
	GY (g)	33.69	30.53	<0.0001	1.58	23.9

NIL^ZS^ and NIL^MY^ are near isogenic lines carrying homozygous *RFT1* alleles from ZS97 and MY46, respectively. Twenty NIL^ZS^ lines and 20 NIL^MY^ lines of the R1 population were grown in Hangzhou, using a randomized complete block design with two replications. HD, heading date. NP, number of panicles per plant. NGP, number of grains per panicle. TGW, 1000-grain weight. GY, grain yield per plant. ^a^ *A*, additive effect of replacing a MY46 allele with a ZS97 allele. ^b^ *R* ^*2*^, proportion of phenotypic variance explained by the QTL effect.

### Effects of the *RFT1*^ZS97^ transgene

The *RFT1* allele from the *indica* rice cultivar ZS97 was transformed into the *japonica* variety ZH11. Genotypes of the transgenic plants were determined using marker Hyg for hygromycin phosphotransferase gene^27^ and Si2926 for *RFT1*. Two types of populations were constructed and used to test the effect of *RFT1* ^ZS97^ transgene.

First, one T_2_ family holding no transgene and five T_2_ families carrying homozygous transgene were produced from independent T_1_ plants. Together with the recipient line ZH11, they were grown in 2011 in Hangzhou and measured for HD and PH. While no significant difference was observed between ZH11 and the transgenic family carrying no transgene, highly significant effects (*P* < 0.0001) were detected for the five T_2_ families carrying homozygous transgene (Table 4). Introduction of the *RFT1* ^ZS97^ allele into ZH11 resulted in delaying HD by 10.7–13.8 d and increasing PH by 6.6–16.5 cm.

**Table 4:** Effect of the *RFT1* ^ZS97^ transgene on heading date and plant height.

Genotype	*n*	Heading date (d)			Plant height (cm)		
		mean ± *sd*	Increase	*P*	mean ± *sd*	Increase	*P*
*RFT1* ^ZS+^	93	82.4 ± 1.3	13.8	4.5 × 10^−73^	128.4 ± 6.0	16.5	9.6 × 10^−22^
*RFT1* ^ZS+^	70	82.3 ± 2.3	13.8	4.3 × 10^−43^	127.8 ± 6.1	16.0	9.5 × 10^−19^
*RFT1* ^ZS+^	91	79.9 ± 0.6	11.3	2.3 × 10^−87^	119.8 ± 4.5	8.0	1.7 × 10^−11^
*RFT1* ^ZS+^	95	79.2 ± 0.9	10.7	2.8 × 10^−74^	121.0 ± 4.0	9.2	2.2 × 10^−16^
*RFT1* ^ZS+^	100	79.3 ± 0.8	10.8	1.1 × 10^−83^	118.5 ± 3.9	6.6	1.0 × 10^−10^
*RFT1* ^ZS−^	91	68.4 ± 1.9	−0.2	0.6911	113.1 ± 3.0	1.3	0.0900
Zhonghua 11	20	68.6 ± 1.2			111.9 ± 3.0		

The five *RFT1* ^ZS97+^ T_2_ families were derived from five independent T_1_ plants carrying homozygous *RFT1* ^ZS97^ transgene. The *RFT1* ^ZS97−^ T_2_ family was derived from a T_1_ plant carrying no transgene. *n*, number of plants. The Student’s *t*-test was used to generate *P* values.

Second, a T_2_ population was derived from a heterozygous T_1_ plant, consisting of 23, 14 and 24 plants carrying no transgene, homozygous transgene and heterozygous transgene, respectively. The 61 T_3_ families were grown in 2012 in Hangzhou and measured for HD, PH and the four yield traits. No significant effect was detected on NGP (*P* = 0.2309), but highly significant effects (*P* < 0.01) were detected for the five other traits with the transgene always having enhancing effects (Table 5). Major effects were detected on HD, PH and TGW, explaining 88.1, 67.3 and 46.9% of the phenotypic variance (*R* ^*2*^), with a *RFT1* ^ZS97^ allele increasing the trait values by 6.12 d, 4.12 cm and 0.82 g, respectively. On the other hand, the effect detected for NP was less important, having an *R* ^*2*^ of 8.7% and an additive effect of 0.37.

**Table 5:** Effects of *RFT1* ^ZS97^ in a segregating population derived from a heterozygous T_1_ plant.

Year	Trait	Phenotypic mean			*P*	*A* ^a^	*D* ^b^	*D*/*[A]* ^c^	*R* ^*2*^(%)^d^
		*RFT1* ^ZS−^	*RFT1* ^ZS+^	*RFT1* ^H^					
2012	HD (d)	65.02	77.25	72.42	<0.0001	6.12	1.28	0.21	88.1
	PH (cm)	101.64	109.88	106.76	<0.0001	4.12	1.00	0.24	67.3
	NP	8.58	9.32	8.99	0.0011	0.37	0.04	0.10	8.7
	NGP	101.76	99.11	101.61	0.2309				
	TGW (g)	25.88	27.53	26.64	<0.0001	0.82	−0.06	−0.08	46.9
	GY (g)	18.66	21.38	20.28	<0.0001	1.36	0.26	0.19	12.0
2013	HD (d)	69.39	77.22	n.a.	<0.0001	3.91	n.a.	n.a.	73.2
	NP	10.39	10.86	n.a.	0.0706				
	NGP	69.90	97.36	n.a.	<0.0001	13.73	n.a.	n.a.	65.4
	TGW (g)	24.42	25.04	n.a.	<0.0001	0.31	n.a.	n.a.	24.0
	GY (g)	22.27	24.86	n.a.	<0.0001	1.30	n.a.	n.a.	71.8

*RFT1* ^ZS+^ and *RFT1* ^ZS−^ are positive and negative homozygous transgenic lines, respectively. *RFT1* ^H^ are segregating lines derived from the *RFT1* ^ZS+^/*RFT1* ^ZS−^ heterozygotes. The numbers of lines tested were 14 for *RFT1* ^ZS+^, 23 for *RFT1* ^ZS−^, and 24 for *RFT1* ^H^. They were grown in Hangzhou using a randomized complete block design with two replications. HD, heading date. PH, plant height. NP, number of panicles per plant. NGP, number of grains per panicle. TGW, 1000-grain weight. GY, grain yield per plant. ^a^ *A*, additive effect of replacing a Zhonghua 11 allele with a ZS97 allele. ^b^ *D*, dominance effect. ^c^ *D*/[*A*], degree of dominance. ^*d*^ *R* ^*2*^, proportion of phenotypic variance explained by the QTL effect. n.a., not available.

Selfed progenies of the 37 homozygous T_3_ families were grown in 2013 in Hangzhou and measured for HD and the four yield traits. No significant effect was detected on NP (*P* = 0.0706), but highly significant effects (*P* < 0.0001) were detected on the four other traits with the transgene always having enhancing effects (Table 5). Major effects with *R* ^*2*^ of 73.2, 65.4, 24.0 and 71.8% were detected on HD, NGP, TGW and GY, respectively. Results obtained from the T_2:3_ and T_2:4_ segregating populations and the T_2_ homozygous families could reach a general agreement that the *RFT1* allele from the *indica* rice ZS97 has a pleiotropic effect to delay heading, increase plant height and enhance yield potential in the genetic background of the *japonica* rice ZH11. This was also accordance with the effect detected in the R1 population in which *RFT1* alleles from *indica* rice ZS97 and MY46 were segregated in an isogenic background, showing that the *RFT1* ^ZS97^ allele has an important effect of enhancing grain yield, increasing plant height and delaying heading.

## Discussion

Recent studies have shown that flowering time genes play important roles in controlling grain yield in rice^9–17^. In the present study, pleiotropism of flowering genes on yield traits was newly found for one of the two florigens genes in rice, *RFT1* having no response to photoperiod. As compared with the *RFT1* alleles from the *indica* rice MY46 and *japonica* rice ZH11, the *RFT1* allele from the *indica* rice ZS97 delayed heading, increased plant height and enhanced grain productivity.

In all the three year’s experiments using NILs of the rice cross ZS97/MY46 (Tables 2 and 3), *RFT1* showed significant effects on grain number, grain weight and grain yield with the enhancing allele derived from ZS97. Major effects were detected in all the three years on grain weight, with the additive effects and *R* ^*2*^ ranging as 0.64–0.95 g and 36.0–79.8%, respectively. The effects detected on grain number were more variable, changing from a major effect with an additive effect of 18.36 and *R* ^*2*^ of 55.7% in 2010 to a minor effect with an additive effect of 3.30 and *R* ^*2*^ of 8.1% in 2013. In the two year’s experiments using segregated transgenic lines (Table 5), the effects detected on grain weight and grain yield were always significant, but the effect found for grain number was significant in 2013 and insignificant in 2012. It could be concluded that *RFT1* plays an important role in the genetic control of grain yield in rice, but its effects on the yield component traits may vary depending on the genetic backgrounds and environmental conditions. This was similar to the variation of the pleiotropic effect of *Ghd7* ^10^, suggesting that dependence of the pleiotropic effects of flowering genes on the genetic background and environmental conditions could be a common occurrence.

It has been reported that yield potential of cultivated rice in relation to ecogeographical adaptation is largely defined by the allelic combinations of *Ghd7*, *Ghd8* and *Hd1* ^13^. The importance of these three genes in determining grain yield in rice has also been shown in many other studies^9–12, 14, 15, 18–20^. It is known that *Ghd7* and *Ghd8* are two key regulators of the *Ehd1*-*RFT1* pathway^7, 8^ and *Hd1* acts as an *Ehd1* repressor through interaction with the Ghd7 protein^21^. Whether the important role of *RFT1* in heading date influencing yield traits in rice relies on the functions of *Ghd7*, *Ghd8* and *Hd1*, or *vice versa*, remains to be clarified.

*RFT1* has a high functional polymorphism^26, 28^ and the effects of *RFT1* are insensitive to day length. This is favourable for evolving a wide adaptation of cultivated rice to various ecogeographical conditions. While early-heading alleles of *RFT1* play a dominant role in high latitude under NLD conditions^29^, the late-heading alleles are favourable in low latitude where short photoperiodic conditions continue throughout the year but long-duration cultivars are required^30^. In rice breeding, the late-heading allele could be utilized for increasing yield potential when the growth duration is not a limitation, and the early-heading allele may be advantageous when the constraint comes in multiple season-cropping systems and in the northernmost region of rice cultivation^17^.

In cloning QTLs for agronomically important traits in rice, map-based cloning has been the most common approach in which a prerequisite is the validation and fine mapping of the target QTL using NIL populations. Classically, NILs are developed through multiple backcrossing and contain a small introgressed fragment in the genetic background of the recurrent parent. More recently, a new type of NILs which are derived from a single recombinant inbred line (RIL) and segregate the target QTL region in an inbred background of the parental mixture has become more and more used^31, 32^. In the present study, an advanced approach of using this type of NILs for QTL fine mapping was applied. As a result, a QTL controlling heading date, plant height and yield traits in rice was placed at the *RFT1* locus. The fine mapping started from the identification of an RH carrying a 7.3-Mb heterozygous segment in a previous study^23^ and ended in the landing of a QTL at one gene locus using two NIL populations in the present study. In addition to the simple and convenient population development described previously^22^, one major advantage of using this new approach is the small sample size required for phenotyping. Usually, phenotyping in QTL analysis only require dozens of lines for each genotypic group.

Two things are especially worthy of mention in using this new approach. One is the production of a segregating population from a single RH plant. In earlier studies, an NIL population was usually derived from a heterogeneous inbred family^33^ or residual heterozygous line (RHL)^34^ which consisted of a few individuals of one RIL. Different plants of an RHL could be segregated as paternal homozygous, maternal homozygous and heterozygous for the target QTL, and the heterozygous segment in the vicinity of the target QTL may not be identical in different plants. Variation may also present in the genetic background. By contrast, a single RH plant has an identical genotype, thus the population derived would be more consistent in the targeted segregating region and more uniform in the genetic background. Even more importantly, the production of a segregating population from a single RH plant could enable the background homogeneity to naturally increase with the generation advancement, thus the genetic noise is decreased automatically.

One other thing worthy of mention is the mapping of QTLs using a sets of NIL populations derived from SeqRHs. In a given population under analysis, presence and absence of a QTL in the segregating region is inferred by significant and insignificant phenotypic variation among different genotypic groups. When a QTL was detected in two or more populations with overlapped segregating regions, the QTL could be located in the common segregating region^22^. When a QTL was only detected in one of two or more populations with overlapped segregating regions, the common segregating regions could be ruled out from the candidate QTL region (the present study). When two or more QTLs were detected in populations with no overlapped segregating regions, different QTLs could be separated^35, 36^. In conclusion, the use of SeqRHs for QTL fine-mapping is advantageous in increasing the efficiency and efficacy of population construction, phenotyping and the dissection of linked QTLs.

## Methods

### Field experiments

The rice populations were grown in the experiment stations of the China National Rice Research Institute located in either the Fuyang Region (120.0 E, 30.1 N) of the Hangzhou City, Zhejiang Province, or the Lingshui County (110.0 E, 18.5 N), Hainan Province, China. Day length in Hangzhou and Lingshui corresponds to NLD and NSD conditions, respectively^19^. A planting density of 16.7 cm × 26.7 cm was used for all the trials. Normal agricultural practice was employed in field management.

All the yield trials were conducted in Hangzhou. Seeds were sown in paddy filed and raised in wet-bed condition for 23–26 days before being transplanted (Supplementary Table S2). From transplanting to harvesting, irrigation was applied to maintain a well-watered condition except that the water was drained for five days at the maximum tillering stage and before harvesting. The experiments followed a randomized complete block design with two replications. In each replication, 12 plants per line were planted in one row except that three rows of 12 plants were used for the two populations tested in 2013. Climatic data for the growing season in 2011 and 2013 were presented in Supplementary Table S3.

In 2010 and 2012 when all the three genotypic groups were used, HD and PH was recorded for individual plants and averaged for each replication. At maturity, panicles were harvested from the middle ten plants and measured for the yield traits, including NP, NGP, TGW and GY. In 2011 and 2013 when only the homozygous lines were tested, HD was recorded for each replication when 50% of the plants in each line were at heading. At maturity, panicles were harvested from middle five plants and measured for the four yield traits except that GY was estimated based on the middle 30 plants in 2013.

### Phytotron experiments

To determine the photoperiodic response of *qHd6*, two pairs of ZS97 and MY46 homozygous lines was selected from the TF6-15 population and tested in controlled chambers. They were grown under SD (10 h light/14 h dark, 12 h 28 °C/12 h 23 °C) and LD (14 h light/10 h dark, 12 h 28 °C/12 h 23 °C) conditions, respectively. Ten plants per line were used and HD was scored for each plant.

### DNA marker analysis

Total DNA was extracted following the method of Zheng *et al*.^37^. PCR amplification was performed according to Chen *et al*.^38^. The products of the DNA markers were visualized on 6% non-denaturing polyacrylamide gels using silver staining. All of the SSR markers were selected from the Gramene database (www.gramene.org). Eight InDel markers (from Si2925 to Si2950 in Supplementary Table S4) were designed using Oligo Primer Analysis Software Version 7.0 (Molecular Biology Insights, Inc.) based on the sequence differences between ZS97 and MY46 detected by the whole-genome re-sequencing.

### Sequence analysis and complementation test

Using primers RFT1-A and RFT1-B (Supplementary Table S4), two fragments overlapped and jointly covering the entire *RFT1* gene region were amplified from ZS97 and MY46, respectively. They were purified and ligated into pGEM®-T Easy Vector, followed by transformation into JM109 high-efficiency competent cells (Promega). Positive clones were selected and sequenced. Nucleotide sequence and the predicted amino acid sequence between ZS97 and MY46 were compared.

The two inserts originated from ZS97 were cut and ligated into the binary vector pCAMBIA1300, with the RFT1-A fragment digested with *Eco*R I and *Kpn* I and the RFT1-B fragment with *Kpn* I and *Sal* I. The integrated gene fragment was 6,523 bp in length, containing 2,309 bp upstream of the transcription start site, 1,391 bp coding region and 2,823 bp downstream of the termination site. The construct was introduced into the *Agrobacterium tumefaciens* strain EHA105 and transferred into the *japonica* variety ZH11, using the method reported by Hiei *et al*.^39^. The empty vector was also transformed into ZH11 as a control.

Genotypes of the transgenic plants were determined by PCR amplification of the genomic DNA using primer Hyg for hygromycin phosphotransferase gene^27^ and Si2926 which was designed according to a 70-bp insert/deletion in intron 1 of the *RFT1* gene. Five independent T_2_ families carrying homozygous *RFT1* ^ZS97^ transgene were selected and used for testing the effect of the transgene on HD and PH. One more T_2_ family which was segregated for the transgene was identified, from which T_2:3_ and T_2:4_ populations were derived for testing the genetic association between the transgene and HD, PH and yield traits.

### Statistical analysis

In the replicated field trials, two-way ANOVA was conducted to test the phenotypic differences among different genotypic groups in each population. The analysis was performed with SAS procedure GLM^40^ as described previously^41^. Given the detection of significant difference (*P* < 0.05), the same model was applied to estimate the genetic effect and the proportion of phenotypic variance explained.

## Electronic supplementary material

Supplementary figures and tables
