# Supplementary material for: Rice Flowering Locus T 1 plays an important role in heading date influencing yield traits in rice
Source: Sci Rep. 2017 Jul 7;7:4918. doi: 10.1038/s41598-017-05302-3 (PMC5501849; doi:10.1038/s41598-017-05302-3)
Supplement: Supplementary file 1 — Supplementary figures and tables [file 41598_2017_5302_MOESM1_ESM.doc]

*Scientific Reports*

***Rice Flowering Locus T 1* plays an important role in heading date influencing yield traits in rice**

Yu-Jun Zhu, Ye-Yang Fan, Kai Wang, De-Run Huang, Wen-Zhen Liu, Jie-Zheng Ying & Jie-Yun Zhuang

**Supplementary Figure S1. Fourteen residual heterozygotes selected from 332 F_9_ plants in 23 F_8:9_ families derived from an F_7_ plant of the *indica* rice cross ZS97/MY46.**

**Supplementary Figure S2. Variation of the ZS97*^RFT1^* allele as compared with the MY46 allele.**

**Supplementary Figure S3. Comparison of amino acid sequences of the RFT1 protein between MY46 and ZS97.**

| **Name** | **Heterozygous region A^a^** | **Length (kb)** | **Heterozygous region B^b^** | **Length (kb)** |
| --- | --- | --- | --- | --- |
| 6-1 | RM510–RM19410 | 82.6 | RM587–RM19417 | 686.3 |
| 6-2 | RM4923–RM19410 | 739.6 | RM19350–RM19417 | 1011.4 |
| 6-3 | RM4923–RM19410 | 739.6 | RM19350–RM19417 | 1011.4 |
| 6-4 | RM4923–RM225 | 1242.3 | RM19350–RM6917 | 2100.6 |
| 6-5 | RM510–RM225 | 585.2 | RM587–RM6917 | 1755.9 |
| 6-6 | RM3414–RM225 | 534.9 | RM510–RM6917 | 1236.2 |
| 6-7 | RM19410–RM6119 | 1514.0 | RM3414–RM314 | 1962.6 |
| 6-8 | RM4923–RM3414 | 707.4 | RM19350–RM19410 | 947.0 |
| 6-9 | RM6119–RM314 | 416.7 | RM1163–RM111 | 907.3 |
| 6-10 | RM6119–RM402 | 1972.1 | RM1163–RM19652 | 2380.2 |
| 6-11 | RM314–RM19784 | 4593.7 | RM6119–RM19795 | 5137.5 |
| 6-12 | RM111–RM19784 | 4341.2 | RM314–RM19795 | 4720.9 |
| 6-13 | RM111–RM19784 | 4341.2 | RM314–RM19795 | 4720.9 |
| 6-14 | RM276–RM19744 | 2199.9 | RM253–RM19784 | 4012.5 |

**Supplementary Table S1. Fourteen residual heterozygotes in the F_9_ generation of the *indica* rice cross ZS97/MY46.** ^a^Heterozygous region A, the segment covered by the heterozygous marker loci. ^b^Heterozygous region B, the segment including the heterozygous region and the flanking cross-over regions.

| **Rice population** | **Year** | **Sowing date** | **Transplanting date** |
| --- | --- | --- | --- |
| NIL sets R1 & R18 | 2010 | 25 May | 17 June |
| NIL set R1 | 2011 | 24 May | 18 June |
| Transgenic population T3 | 2012 | 28 May | 23 June |
| NIL set R1 & Transgenic population T4 | 2013 | 22 May | 16 June |

**Supplementary Table S2.** **Sowing and transplanting date of the four yield trials conducted in Hangzhou.**

| **Parameter** | **2011** | **2013** |
| --- | --- | --- |
| Average daily temperature (°C) | 24.6 | 26.0 |
| Average daily temperature min (°C) | 14.4 | 13.6 |
| Average daily temperature max (°C) | 32.4 | 35.9 |
| Average daily photoperiod (h) | 13.1 | 13.1 |
| Average daily solar radiation (MJ/m^2^) | 11.3 | 13.1 |

**Supplementary Table S3. Climatic data collected from 9 May to 31 October in 2011 and 2013.**

| **Name** | **Forward (5′−3′)** | **Reverse (5′−3′)** |
| --- | --- | --- |
| Si2925 | TTGGCTAGCTTAACCTTCC | GGCCATGTCAAATTAATAACCTC |
| Si2926 | TACGGATCATATCTTGGATGC | TAACATATCTGATCGGTGCCTA |
| Si2927 | AACATGAGAAAATAACTTGCACAT | TCATTCTGATAGCTTTGTCCC |
| Si2933 | AGTACTACATACCCCTATCCAC | AATATATGTATAGACCACGCGCACT |
| Si2936 | AAACTTAAAGAAATTTGACTATGAAA | AACAAGATCTGATCAACTTCAC |
| Si2940 | TGCACACTCATTTCAAGATTCAACT | GTCAAACTTGGCATGTACGGTCT |
| Si2944 | AAACCACAAGATTAGGCTCTAAGT | AAGTGAGGGAAACCACATTCTAC |
| Si2950 | AGGCACTCACAACCATAAC | TAGTACATGACAATGGAGCTATC |
| RFT1-A | ACGCGTCGACGTGATTCGGTGGGCGTAACTG | GGTACCAGGAATATCGGTGACCAG |
| RFT1-B | CCTGGTACCACTGGAGCAACATT | GCCAAGCAGAATCTGAATCC |
| Hyg | GTTTATCGGCACTTTGCATCG | GGAGCATATACGCCCGGAGT |

**Supplementary Table S4.** **Primer sequences.** Hyg was used following Liu *et al*^27^ and the others were developed in the present study.
